# The Global Initiative for Asthma 2019 recommendation for mild asthma – A critique

**DOI:** 10.4102/safp.v62i1.5104

**Published:** 2020-03-05

**Authors:** Elvis M. Irusen

**Affiliations:** 1 Division of Pulmonology, Department of Medicine, Faculty of Medicine and Health Sciences, Stellenbosch University, Stellenbosch, South Africa

**Keywords:** GINA 2019, mild asthma, regular ICS + LABA combination, critique

## Abstract

Recognising that mild asthmatics are at risk of exacerbations and mortality, the Global Initiative for Asthma (GINA) issued an updated strategy in 2019. This was premised on two studies culminating in their recommendation that mild asthma should be treated by using a combination of a rapid and long-acting beta 2 agonist and an inhaled corticosteroid (ICS) administered as required. Their rationale is, however, debatable, as the studies actually showed that regular daily ICS administration was more effective for a number of asthma control endpoints. A patient-driven treatment strategy is also questionable, as there are a number of concerns about behaviour of patients suffering from asthma and perception of airway narrowing that should trigger medication intake but in fact does not do so. These deficiencies also influence a similar maintenance and reliever treatment (MART) approach that would be suboptimal. Intermittent ICS regimens are also inferior when compared to regular treatment. Not all asthmatics respond to the same dose of ICS. The best way to manage asthma is by adopting a step-up ICS approach, to encompass varying disease severity, with a long-acting beta agonist taken on a daily basis, ideally in a single combination inhaler.

## Introduction

Mild asthmatic subjects experience exacerbations and are at risk of mortality. This was the startling revelation documented in the latest Global Initiative for Asthma (GINA) strategy document, which focusses largely on mild asthma.^1^ There are three components to consider in the management of asthma: control, severity and the risk of exacerbations. Asthma control refers to the manifestations of asthma and to the extent that they can be ameliorated by treatment. Severity is often assessed retrospectively by the amount and the number of medications to bring asthma under control; the higher the dose of the inhaled corticosteroid (ICS) and multiplicity of agents, the greater the severity. The risk of exacerbations is primarily influenced by the ability to control the disease process, that is, better symptom control diminishes the risk. However, other factors for increased risk include non-prescription of ICS, poor adherence and inhaler technique, co-morbidities, persistent allergen exposure, smoking and previous admission to an intensive care unit.^1^

To address the specific concerns of mild asthma, members of the science committee of GINA embarked on research studies that culminated in their recommendation that the preferred option is a rapid and long-acting beta 2 agonist (LABA) with concomitant ICS taken only when needed as the best way to obviate the risks of asthma.^2,3^ A critical analysis of the research that informed this recommendation reveals that it was in fact not the best strategy. That re-evaluation in the context of mild asthma and other therapeutic options that have been studied previously will be appraised.

## The concept of mild, intermittent asthma

It appears incomprehensible that mild asthmatics should be subject to the major risks described above. In surveys including over 400 000 asthmatic subjects, the exacerbation rate amongst mild asthmatics (step 1 GINA) was similar to that for mild and moderate chronic persistent asthma (steps 2–3).^4,5^ In one mortality report, 33% of child deaths^6^ and in another given by the Royal College of Physicians of London,^7^ 9% of all mortalities occurred amongst mild asthmatics. Clearly, a disease that puts one at risk for acute attacks and possible deaths should not be considered ‘mild’. Thus, its severity grading should be elevated to chronic persistent, and it should get the appropriate treatment for that category. There are further reasons to support this stance. It has been well-established that patients underestimate the severity of their condition and doctors equally, unfortunately, do the same, putting patients into a lower severity stage than that to which they belong.^8,9^ In addition, it has been shown that mild asthmatics also experience airway remodelling, especially if the risk factors for exacerbation (mentioned earlier) are present.^10^ This refers to the increase in the thickness of the sub-epithelial layer of the bronchi because of chronic airway inflammation. The consequence is a degree of ‘fixed obstruction’ (an irreversible component) as well.

It would also be expected that mild asthmatics would have minimal symptoms and negligible impact on their quality of life (QOL). However, in a large multi-country study, such patients, in reality, experienced significant nocturnal symptoms and work and activity impairment.^11^ The reason for the underassessment of the severity is related to both patient- and physician-related factors.^9^ Patients are well-known to underplay their symptoms and impact of asthma.^12^ Doctors in turn, perhaps because of limited time for consultations, do not assess asthma in a structured manner.^9,13^ Important aspects of deficits of the impact of asthma in terms of both control and QOL are overlooked.

The major change in the GINA 2019 strategy is that short-acting *β*_2_ agonists (SABA) used *pro re nata* (prn) are no longer recommended as the primary agents for episodic symptoms or rescue. Instead, this has been substituted by rapid-acting, long-acting *β*_2_ agonists (LABA-formoterol) with a steroid, budesonide, in a combination inhaler, to be taken only when symptomatic. The GINA science committee also states that this is the preferred recommendation. One needs to point out immediately that there are no data to support this regimen as ‘preferred’ nor is there regulatory approval anywhere in the world for this indication. There has been no study of patient preferences that informed this change. Rather, the suggestion was informed by the two studies that were conducted and reported on by members of GINA themselves (Symbicort Given as Needed in Mild Asthma [SYGMA] 1 and 2).^2,3^ These studies enrolled mild asthmatics who had been using *β*_2_ agonists alone or had been using low-dose ICS + SABA. They were then randomised to either SABA only or quick-acting LABA + ICS combination treatment to be taken as required or to a treatment involving budesonide to be taken twice daily (SYGMA 1 employed all three regimens whilst SYGMA 2 utilised only the latter two options). The ICS regimens were superior to SABA alone for asthma control in terms of reduction in symptoms. Interestingly, the exacerbation rates improved with the ICS regimens, compared to SABA, but were not statistically significantly different whether taken as required or on a regular basis. However, asthma attacks were not completely abolished (see Table 1).

**TABLE 1:** A comparison of asthma control in the Symbicort Given as Needed in Mild Asthma studies versus Gaining Optimal Asthma Control.

Parameter	Budesonide – formoterol prn	Daily budesonide	Superiority of daily budesonide	GOAL stratum 1 – steroid-naive*
% Well controlled week/patient	37.5	47.7	27%	80%
Annualised exacerbation/patient	0.14	0.15 (NS)	-	0.07
ACQ: 5% of patients				
Improved	42.3	48.4	14%	-
Worsened	13.7	10.3	25%	-
No Change	44	41.3	-	-
AQLQ: % meeting CID	0	0	-	80%
Lung function improvement: pre-bronchodilator	65 mL	120 mL	*p* < 0.001	-

ACQ, Asthma Control Questionnaire; AQLQ, Asthma Quality of Life Questionnaire; NS, not significant; CID, clinically important difference; GOAL, Gaining Optimal Asthma ControL; prn, *pro re nata*.

* , This is an indirect comparison.

It must be further pointed out, that regular ICS was superior in a number of other outcomes and the authors of both the papers conceded this unequivocally! It is therefore a little surprising that in the GINA document, they chose the as-needed LABA + ICS as the preferred regimen. One of the reasons that they rationalised was that reasonable (but inferior) control was acceptable because the steroid load would be lower (mean of 90 *µ*g in the prn group vs. 320 *µ*g in the regular group). There appears to be no credence to this argument as 320 *µ*g budesonide is exceedingly safe (the therapeutic threshold is 800 *µ*g beyond which systemic side effects are possible).^14^ Thus, if the patient in the regular ICS group exceeded this dose, one could understand the safety issue; at a dose ≤ 400 *µ*g, this is not a consideration.

The multiple other benefits and superiority of regular ICS are shown in Table 1. The first point to be noted is the improvement in the pre-bronchodilator lung function. When corticosteroids are initiated in patients with asthma, there is an improvement in the pre-bronchodilator forced expiratory volume in the first second (FEV1).^2^ That is, as inflammation subsides, there is spontaneous bronchodilatation. This is how the efficacy of ICS should be monitored. Unfortunately, currently, this is usually difficult because many patients are already on ICS + LABA combinations, and the bronchodilator makes it difficult to check the ICS response. In SYGMA, those subjects randomised to prn ICS + LABA had lower pre-bronchodilator lung function, compared to the regular ICS group. This implies that airway inflammation was inadequately controlled, with the FEV1 difference at 1 year being approximately 55 mL. There is a real concern that this suboptimal control of airway inflammation could result in airway remodelling over time – fixed asthma – that simulates the spirometry seen in chronic obstructive pulmonary disease subjects.

Secondly, the results of the QOL questionnaires reveal that over 50% of subjects in the prn group had no improvements or worsening in the QOL. This implies that this group actually needed more ICS than what was prescribed. Notably, for both questionnaires, not a single patient in either regimen (see Table 1) met the criterion for a clinically important difference (CID) > 0.5. This again implies that higher doses of ICS were needed. The SYGMA studies employed fixed regimens. This is an old style of conducting a trial. This was commonplace over two decades ago during the development of ICS; for example; 400 *µ*g beclomethasone dipropionate would be compared to 400 *µ*g fluticasone propionate (FP). Superiority of the latter (as it is an improved ICS with better relative receptor affinity)^15^ was not unexpected, but did this achieve the best clinical outcome, that is, what would 800 *µ*g of FP achieve? The ICS equipotence trials largely ended with the landmark Gaining Optimal Asthma ControL (GOAL) study.^16^ For the first time, medication, salmeterol + FP, was up-titrated until total control of asthma was achieved (one of the qualifying criteria was that a patient would not use salbutamol at all in 7 of the previous 8 weeks of assessment – an extremely rigorous standard). By way of comparison, although admittedly indirect, the stratum 1 (ICS naïve) group of the GOAL study was compared to the SYGMA studies (Table 1). One notes that potentially greater reductions in exacerbation rates are possible, and 80% of patients met the CID in the QOL questionnaire.

## Should inhaled steroids be used as needed (intermittently) or daily?

Corticosteroids exert their beneficial effects for controlling the disease through a number of genomic and non-genomic actions.^17^ One method to assess the efficacy of ICS is to look at the bronchodilator response because as has been suggested earlier, as inflammation decreases, the bronchi spontaneously dilate. In one report, 1600 *µ*g of Budesonide was administered to a subject as a single dose; the Peak Expiratory Flow Rate (PEFR) began increasing over a few hours reaching a peak at 8 h whereafter the PEFR began decreasing back to the baseline.^18^ In the experiment, the anti-inflammatory effect therefore lasted for only 8 hours. Thus, if patients only use ICS intermittently, there is cover for a short time and there may be substantial periods where there is unopposed inflammation in the airways. This is one of the reasons for the potential for exacerbations and remodelling.

Unsurprisingly, therefore, a number of studies prior to SYGMA have unequivocally demonstrated that intermittent ICS is inferior to daily ICS in both children and adults.^19,20^ Intermittent therapy results in satisfactory (but not optimal) asthma control and, in general, only an improvement in the PEFR. Daily ICS administration has been associated with a more comprehensive control of inflammation and improvement of:

- forced expiratory volume in the first second
- fractional exhaled nitric oxide (FeNO) – a biomarker of asthmatic inflammation
- bronchial hyper-responsiveness
- percentage of eosinophils in sputum (reflecting lower airway allergic inflammation)
- symptom-free days
- Asthma Control Test (ACT) scores – a validated simple 5-point questionnaire)^13^
- exacerbation reduction.

Another strategy to treat asthma is maintenance and reliever therapy (MART) that utilises a quick and long-acting ß agonist plus ICS in a combination inhaler.^21^ The patient uses a lower dose of baseline maintenance treatment, and when symptoms arise uses the same LABA + ICS product and not salbutamol. This protocol has been purported to provide superior control. However, when one studies the trials carefully, other observations can be made. In one trial, where MART apparently resulted in superior outcomes, it was noted that patients took an extra inhalation of the LABA + ICS product every single day for symptoms.^22^ This actually implies that patients were taking an extra dose of ICS daily, which was equivalent to a 50% increase in corticosteroid dosage, compared to the group taking ICS plus SABA (for relief) regularly on a daily basis; so one was actually comparing an overall higher ICS regimen – which unsurprisingly resulted in better outcomes.

## Can patients be relied upon to adhere to *pro re nata* (prn) regimens?

Patient-driven treatment (MART or a SYGMA type strategy) is dependent on the patient perceiving symptoms and triggering the intake of medication. There are two problems with this approach. Firstly, asthmatics are quite accustomed to episodic chest tightness and, therefore, they often do not take treatment when they should.^23^ Secondly, this approach implies that the patient can accurately perceive airway closure; this is not true either. It has been shown that patient perception is blunted, and they frequently overlook decreases in airflow.^21^ These factors mean that patients will underdose themselves with the attendant risks. A fundamental problem with MART is that it is dependent on symptoms that are frequently consequent to increased inflammation, and thus treatment is reactive. The effects of these periods of heightened inflammation in the airways are unknown. In contradistinction, regular maintenance treatment is proactive, preventing instances of increased inflammation and potentially decreasing the likelihood of airway remodelling.

## Global Initiative for Asthma recommends that salbutamol should not be used for breakthrough symptoms

Another recommendation in GINA 2019 is that when patients have chest tightness, salbutamol is an option, but the preferred reliever is again the product having the formoterol and budesonide combination. Once again, there is no data to support this strategy. One should ask if it is appropriate that episodic symptoms be treated with LABA + ICS instead of salbutamol. Salbutamol has been used for decades for acute relief in the chronic care of asthmatics. It provides quick relief, and both patients and doctors have confidence in the product. It is quite inexpensive, compared to an LABA + ICS inhaler – thus, if this were to change, it will increase healthcare costs. The recommendation of GINA is based on the experience of overreliance on SABA in treating asthma and underutilisation of ICS.^23,24^ This challenge is better solved with education rather than by using a much more expensive alternative.

In making the reliever option change, GINA, responding to patient behaviour, states that mild asthmatics would rather take treatment intermittently than daily. In the SYGMA studies, daily treatment was superior. Should we change our treatment strategies because of patient behaviour? Hypertensive subjects take medication daily in spite of the absence of symptoms because it represents correct management. Would we give diabetics medication on an intermittent basis? In asthmatics, we should educate and reinforce the messages of appropriate treatment; intermittent treatment that is inferior is not a substitute for not educating patients on the correct way to manage their condition.

## Conclusion

One should note that GINA no longer publishes guidelines but rather makes recommendations – a ‘strategy’ – that allow clinicians to review the information provided and decide on management issues themselves. In following the strategy, the severity and categorisation of asthma needs to be accurately defined. If asthma is truly intermittent – only very occasional symptoms – then SABA alone suffices. If there are more frequent symptoms or exacerbations, then asthma should be classified as chronic and persistent, and the SYGMA studies have shown that in this scenario daily ICSs are superior to prn Formoterol and Budesonide, a fact that the authors themselves have conceded.

One must accept that we do not have robust measures to assess airway inflammation, and our ability to titrate therapeutic interventions are at best a guess based on symptoms and lung function, both unreliable tools. However, physician-directed treatment is better than patient-driven treatment, as there are problems with perception of airway narrowing and patient behaviour in asthma, leading to inadequate treatment. Daily regular ICS therapy, stepping up in dosage dependant on disease severity with the addition of a LABA, has been demonstrated to regulate inflammation better with optimal control endpoints. Addition of the LABA also augments ICS activity allowing a lower ICS dose, thereby decreasing the steroid load.^16^ This is the best approach to prevent exacerbations and to impact the mortality related to asthma which are persistent global concerns.
